# Development, validation, and usage of metrics to evaluate the quality of clinical research hypotheses

**DOI:** 10.1186/s12874-025-02460-1

**Published:** 2025-01-16

**Authors:** Xia Jing, Yuchun Zhou, James J. Cimino, Jay H. Shubrook, Vimla L. Patel, Sonsoles De Lacalle, Aneesa Weaver, Chang Liu

**Affiliations:** 1 College of Behavioral, Social, and Health Sciences, Clemson University, Clemson, SC USA; 2 Patton College of Education, Ohio University, Athens, OH USA; 3 Department of Biomedical Informatics and Data Science, Heersink School of Medicine, University of Alabama, Birmingham, AL USA; 4 College of Osteopathic Medicine, Touro University, Vallejo, CA USA; 5 The New York Academy of Medicine, New York, NY USA; 6 College of Art and Science, California State University Channel Islands, Camarillo, CA USA; 7 Russ College of Engineering and Technology, Ohio University, Athens, OH USA; 8 Department of Public Health Sciences, College of Behavioral, Social, and Health Sciences, Clemson University, 519 Edwards Hall, Clemson, SC 29634 USA

**Keywords:** Clinical hypothesis evaluation, Metrics, Instrument development, Validation, Clinical research, Scientific hypothesis evaluation

## Abstract

**Objectives:**

Metrics and instruments can provide guidance for clinical researchers to assess their potential research projects at an early stage before significant investment. Furthermore, metrics can also provide structured criteria for peer reviewers to assess others’ clinical research manuscripts or grant proposals. This study aimed to develop, test, validate, and use evaluation metrics and instruments to accurately, consistently, systematically, and conveniently assess the quality of scientific hypotheses for clinical research projects.

**Materials and methods:**

Metrics development went through iterative stages, including literature review, metrics and instrument development, internal and external testing and validation, and continuous revisions in each stage based on feedback. Furthermore, two experiments were conducted to determine brief and comprehensive versions of the instrument.

**Results:**

The brief version of the instrument contained three dimensions: validity, significance, and feasibility. The comprehensive version of metrics included novelty, clinical relevance, potential benefits and risks, ethicality, testability, clarity, interestingness, and the three dimensions of the brief version. Each evaluation dimension included 2 to 5 subitems to evaluate the specific aspects of each dimension. For example, validity included clinical validity and scientific validity. The brief and comprehensive versions of the instruments included 12 and 39 subitems, respectively. Each subitem used a 5-point Likert scale.

**Conclusion:**

The validated brief and comprehensive versions of metrics can provide standardized, consistent, systematic, and generic measurements for clinical research hypotheses, allow clinical researchers to prioritize their research ideas systematically, objectively, and consistently, and can be used as a tool for quality assessment during the peer review process.

**Supplementary Information:**

The online version contains supplementary material available at 10.1186/s12874-025-02460-1.

## Introduction

A hypothesis is an educated guess or statement about the relationship between two or more variables [1, 2]. The hypothesis generation process is critical and decisive in determining the significance of a clinical research project or scientific project. Although much progress has been achieved in scientific thinking, reasoning, and analogy [3–8], which are critical skills in hypothesis generation, knowledge about the scientific hypothesis generation process, including how to facilitate the process, especially in a clinical research context, is limited. Many data science researchers believe that secondary data analytic tools can facilitate hypothesis generation [9]. Nevertheless, there is a lack of studies demonstrating the role of a secondary data analysis tool in this process in clinical research. We developed a visual interactive analytic tool for filtering and summarizing large health data sets coded with hierarchical terminologies (VIADS, https://www.viads.info [10]) to filter, compare, summarize, and visualize datasets coded with hierarchical terminologies (e.g., International Classification of Diseases, 9th Revision, Clinical Modification, ICD-9-CM). VIADS can also assist clinical researchers with generating hypotheses. Visual examples of VIADS include hierarchical graphs to show the structure of ICD, bar charts, and 3D plots. Users can obtain expanded information via interactive features, change graph layouts (e.g., small, medium, and large horizontal spacing), zoom in and out, and move, save, and export graphs and their data files.

To put this manuscript in the appropriate context, we provide some background information on the entire project and how we conducted it to elaborate on how this study fits the bigger picture. To explore the clinical researchers’ hypothesis generation processes, we conducted one-on-one study sessions in which researchers (i.e., participants) analyzed the same datasets to generate hypotheses within two hours with or without VIADS [11]. This was a 2 × 2 study design (with and without VIADS by experienced and inexperienced clinical researchers per predetermined criteria). The quality of each scientific hypothesis generated by the participants in the study [12, 13] was assessed by an expert panel using the same metrics. The aggregated quality assessment results, along with the number of hypotheses, the average time, and the number of cognitive events used to generate a hypothesis, were used to compare the hypotheses generated by the participants in different groups [12, 14]. A reliable, generic, and convenient tool is required to have a reliable, consistent, and accurate assessment of the quality of the generated scientific hypotheses [15].

The original purpose of developing metrics is to evaluate the hypotheses generated by the participants in our research project. Furthermore, the validated metrics and instruments can be useful in a broader clinical research context. Researchers can use the instruments to compare and select more valuable and impactful hypotheses to pursue in their research endeavor at an early stage before any significant investment in resources. Furthermore, the instruments can be used during peer review processes for clinical research manuscripts or grant proposals. Traditionally, the peer review process is conducted by human experts, which can be a subjective assessment. Using an explicit, clearly defined, consistent, and comprehensive assessment tool based on metrics can provide a solid foundation for a relatively more objective, consistent, and perhaps more accurate evaluation during the peer review process of clinical research projects. The lack of a significant, meaningful, and impactful hypothesis to start with can make all other aspects of the research projects meaningless, regardless of rigor or validity. Therefore, the development and validation of such metrics play an important role in facilitating the launch of a more impactful research project and conducting a more objective, consistent, and accurate peer-review evaluation. In this manuscript, we introduce the approach we used to develop and validate the metrics, the results of the metrics and instruments, and the preliminary experience of the usage of the metrics. We hope to share the metrics and instruments as potential tools and the methodology we used to develop them with the clinical research community.

## Materials and methods

### Study flow

**Fig. 1:**
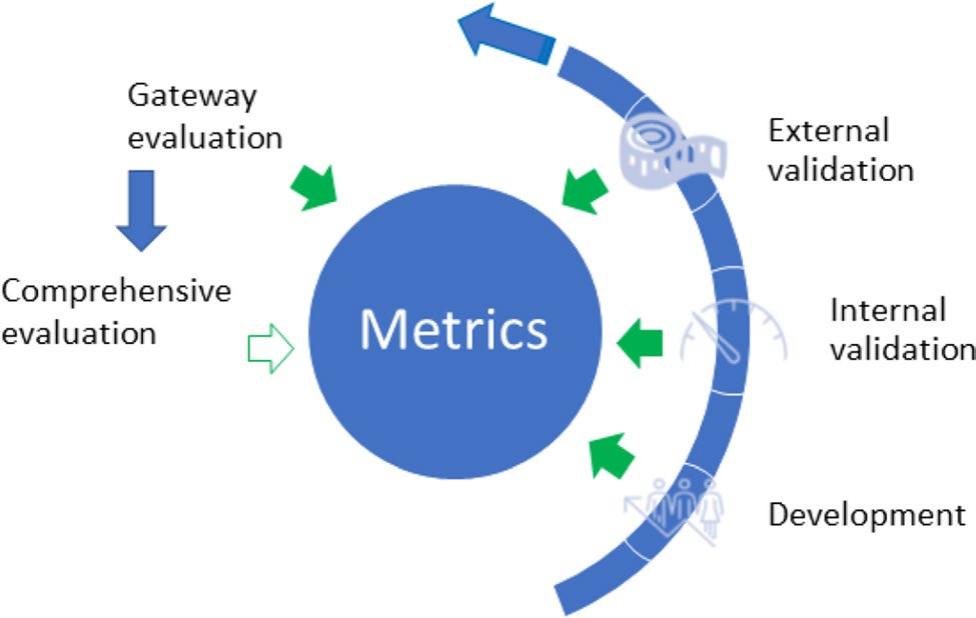
Development, validation, and usage of the metrics to assess the quality of clinical research hypotheses. Blue arrows indicate the development stages of metrics; solid green arrows indicate the feedback incorporated into the metrics from each stage; green hollowed arrow indicates future work

In order to identify the metrics that we can use to assess scientific hypotheses in clinical research, we conducted this study in the following steps: (1) metrics development, (2) internal validation (two layers), and (3) external validation (two experiments, Fig. 1) [16–18] and iterative revisions and refinements of the metrics at each step.

### Metrics development

After several failed literature searches without returning needed results when we aimed to identify the existing metrics to evaluate scientific hypotheses in clinical research, we started conceptualizing and developing the metrics through literature review and initial metrics formulation and development. One author (XJ, a medical informatics researcher) reviewed the clinical research design, clinical research methodology, and clinical trials-related literature [1, 19–29] and drafted the initial metrics.

### Internal validation

Then, two authors (XJ, a medical informatics researcher, and YCZ, a research methodologist) discussed the outlined metrics individually and formulated the initial metrics. They revised the metrics after all confusion and concerns were addressed iteratively. This was the first internal validation layer between two team members. The adjusted metrics were distributed to the research team as anonymous surveys to seek feedback on all evaluation items. This step was conducted in three rounds to incorporate all the feedback received. This step constituted the second layer of internal validation among the entire team. The internal validation processes on the instrument (i.e., the evaluation dimensions, subitems, and scales of subitems) followed a revised Delphi method [30–34], which included transparent and open discussions (via face-to-face meetings, emails, and complementary video conferences) among the research team.

### External validation

After completing the internal validation, an iterative external validation process was conducted by engaging an additional four invited clinical research experts who are external to our team. The criteria to be eligible as a clinical research expert were pre-defined during the design of the research project (please refer to our prior publication for details [11]). The instrument used in the initial external validation is shown in Appendix 1.

**Fig. 2:**
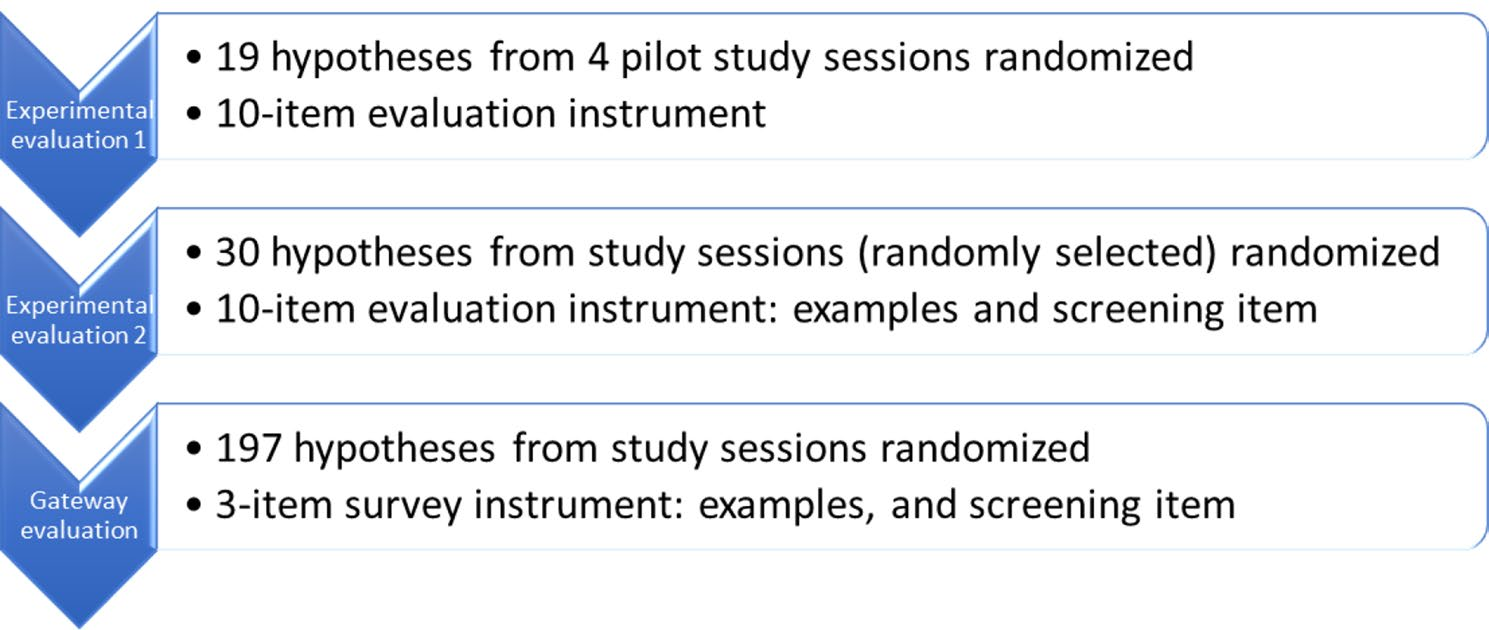
Refinement process of the clinical research hypotheses quality evaluation instrument

The external validation consisted of three steps, (1) initial external validation of the metrics via surveys among expert panel members, (2) two experimental evaluations by using the metrics to assess hypotheses generated during the study sessions, and (3) refinement based on the feedback and results of the experimental evaluations (Fig. 2). A survey (Appendix 2) that served as the medium validation instrument was used among all expert panel members (including three senior consultants from the research team and four external clinical research experts) to obtain feedback, which was incorporated into the final metrics (Table 1 and Appendix 3). A 10-item evaluation instrument was formulated from the development and validation processes. The initial external validation used a revised Delphi method, including transparent discussions via emails and complementary video conferences among the expert panel members.

### External validation: experimental evaluation 1

**Fig. 3:**
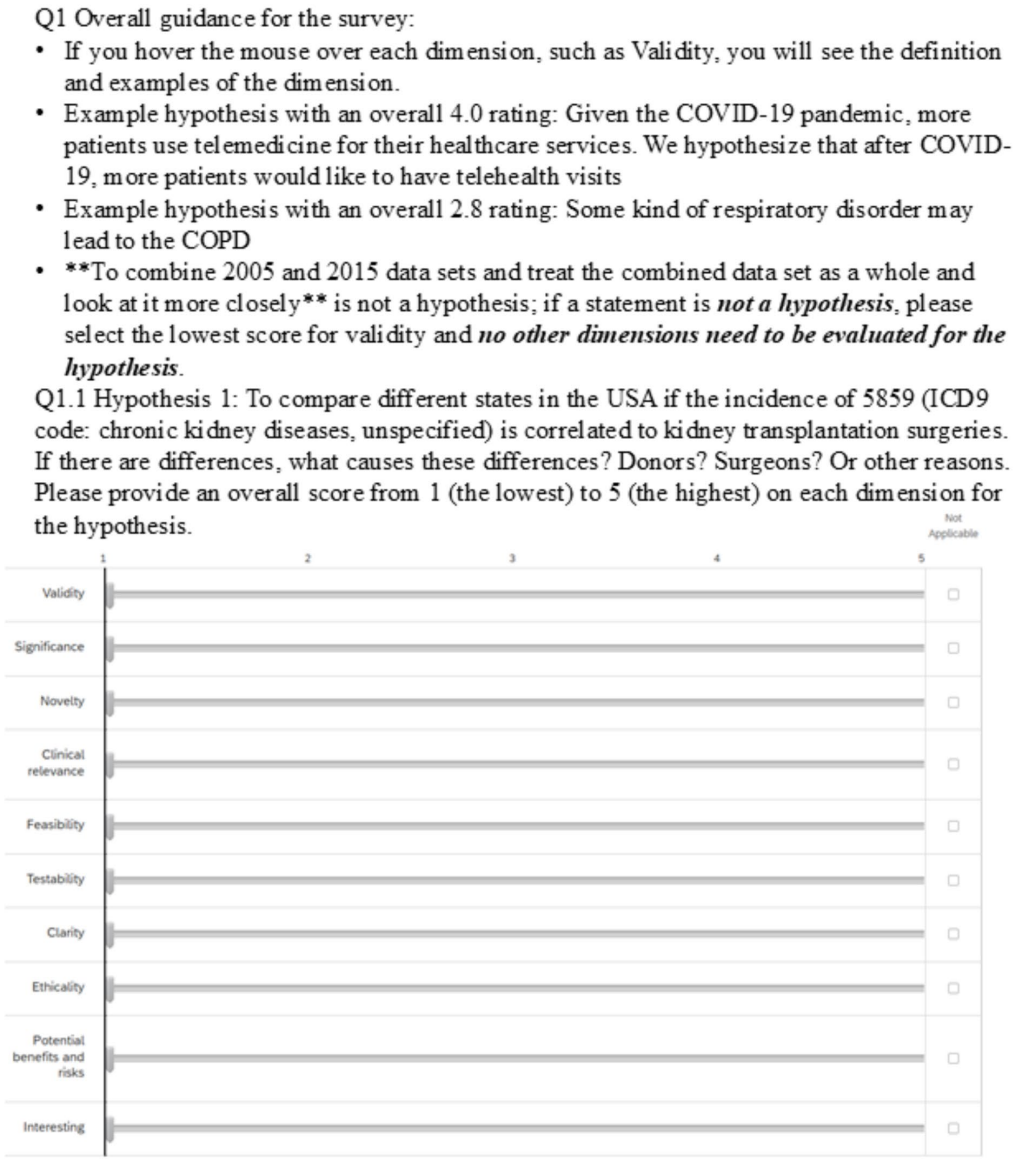
Ten-item evaluation instrument for clinical research hypothesis screening and evaluation

In experimental evaluation 1, we performed validation analysis for the ten evaluation items (without subitems) using 19 hypotheses generated via pilot studies of the research project. These hypotheses were randomly assigned to two Qualtrics surveys (10 and 9 hypotheses per survey). The seven expert panel members are our evaluation team, all of whom have a medical or methodology background with decades of experience working in a clinical research context. They rated all the hypotheses. The inter-rater agreement of the seven experts’ ratings on the 19 hypotheses was analyzed using the intra-class correlation (ICC). We used descriptive statistics to analyze the results of the survey. Based on the mean rating results (i.e., the average rating scores for each hypothesis) from experimental evaluation 1, we identified the best and worst examples of hypotheses, which were used as examples in experimental evaluation 2 for the expert panel members to better calibrate their rating scores in the assessment of the remaining hypotheses. Figure 3 shows the survey we used to conduct experimental evaluations 1 (without the highest and lowest rated examples) and 2 (with the highest and lowest rated examples).

### External validation: experimental evaluation 2

The results of Experiment 1 indicate that Experiment 2 is necessary. Experimental evaluation 2 included 30 randomly selected hypotheses from the study sessions using the 10-item evaluation instrument (Fig. 3). In the instructions, we provided the highest and the lowest rated examples of hypotheses based on the experimental evaluation 1 results and set a screening item: validity. If a statement is not a hypothesis, further evaluation is unnecessary. If three or more experts scored at 1 (lowest rating) in validity for any of the hypotheses, it was removed from the following analysis. ICC analysis was performed to examine the consistency of the seven experts’ ratings on the valid hypotheses using the ten items. The evaluation results, i.e., the quality ratings of hypotheses based on the different evaluation items (metrics) were compared using a paired *t*-test analysis. The test results help us to identify a simpler version of the instrument that can be used reliably and practically to evaluate all hypotheses generated by clinical researchers, i.e., Gateway evaluation in Figs. 1 and 2.

### Instruments used

All steps mentioned above (initial draft metrics development, internal validation, external validation, refinement, and revisions in between the steps) were conducted iteratively using quantitative and qualitative approaches (e.g., Qualtrics surveys, emails, additional phone calls, and virtual conferences). The evaluations of the instrument (with 10 items and 39 subitems), i.e., the validation process before experts used the instrument to conduct the experimental evaluations, including a 5-point Likert scale and three additional options of unable to assess, unnecessary subitem, or use this item only (Appendix 2). The evaluation instrument (with 10 items) used in experimental evaluations 1 and 2 included a 5-point Likert scale and an option of not applicable (Fig. 3). The gateway evaluation and its results are published separately [14, 35]. This study was approved by the Ohio University Institutional Review Board (18-X-192) and Clemson University Institutional Review Board (IRB2020-056).

## Results

**Fig. 4:**
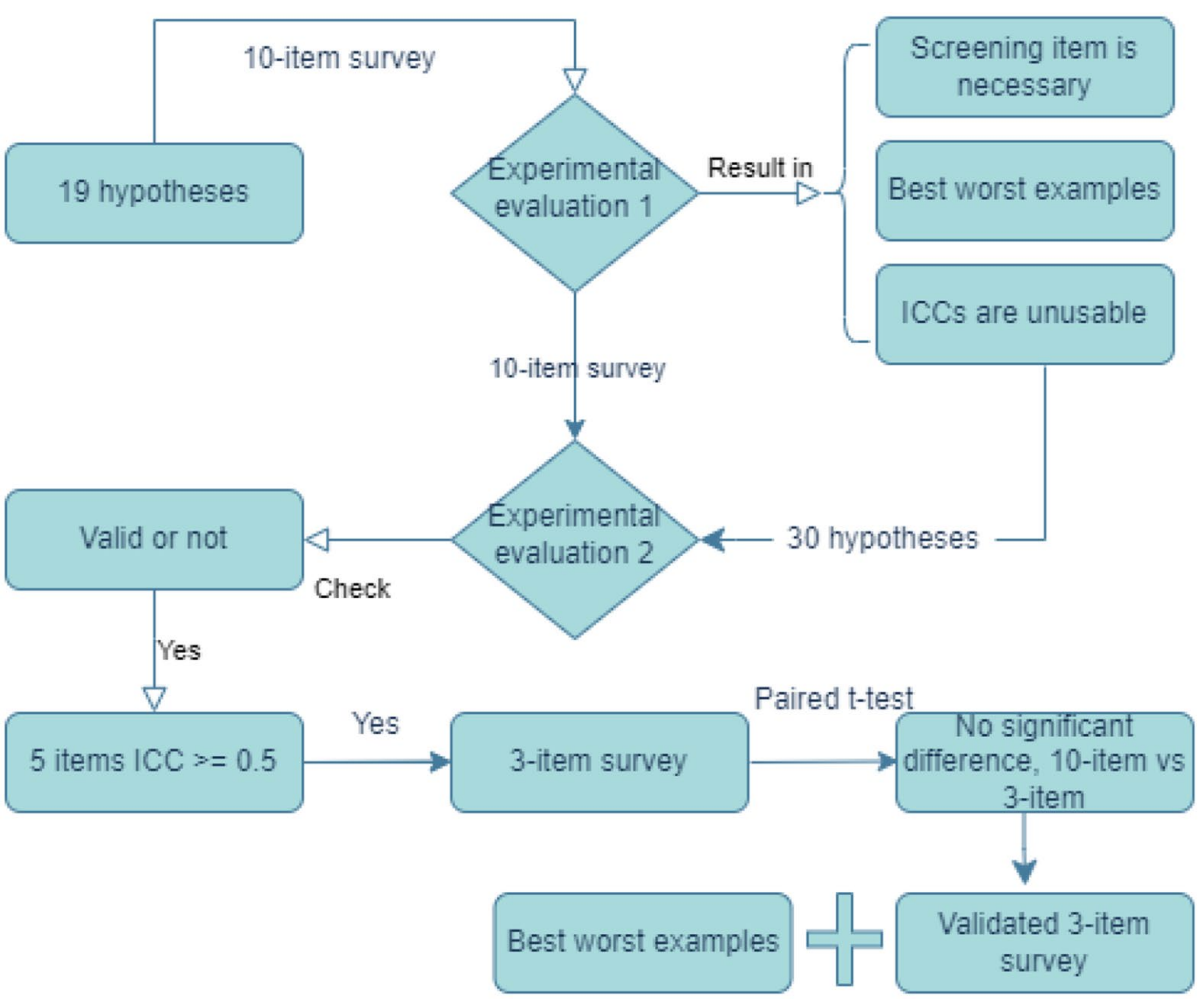
Summary of methods, steps, and corresponding results of development and validation of metrics in assessing the quality of clinical research hypotheses

We present comprehensive (10 items and 39 subitems, Appendix 3) and brief versions (3 items, 12 subitems, Table 1, Appendix 4) of the instrument to assess the quality of clinical research hypotheses and the evidence generated from experimental evaluations. Figure 4 presents the steps used in this study and the corresponding results to provide a summary view of the methods and results. Most measurements for evaluating the quality of clinical research hypotheses from the literature [1, 2, 9, 19–22, 24–29, 36] include the following ten dimensions: ***validity***, ***significance***, ***novelty***, ***clinical relevance***, ***potential benefits and risks***, ***ethicality***, ***feasibility***, ***testability***, ***clarity***, and ***researcher interest level***. We developed 39 sub-items to measure each dimension comprehensively and unambiguously (Table 1). The quality of each item was measured using a 5-point Likert scale. Table 1 shows all ten evaluation items (i.e., dimensions) and subitems and how they were used to evaluate the quality of clinical research hypotheses. Table 2 presents two examples of hypotheses and their quality evaluation results among all evaluators when using the 3-item instrument (Appendix 4).

**Table 1:** Evaluation items and subitems in the metrics used to assess the scientific hypotheses in clinical research

Evaluation items (10)	Subitems (39)	Definition/note
Clarity		
	Clear purposes	The hypothesis is clear in each aspect (i.e., subitems), evaluated on a 5-point Likert scale
	Clear, focused groups	
	Specified variables	
	Specified relationships among variables	
	Overall clear	
Clinical relevance		
	Impact on current clinical practice	To test if the hypothesis has the potential to have a significant impact on each of these aspects (i.e., subitems), evaluated on a 5-point Likert scale
	Impact medical knowledge	
	Impact health policy	
	Overall clinically relevant	
Ethicality		
	No ethical concerns	When conducting a study to test a given hypothesis, there are no ethical concerns (regarding stakeholders and conduction). Consider using *binary options* instead of a 5-point Likert scale
	Trade my place with a participant if eligible	
	Overall, an ethical study to test	
**Feasibility**		
	Regarding needed costs	To test if the hypothesis is feasible regarding the available resources and scope of the work, evaluated on a 5-point Likert scale
	Regarding needed time	
	Regarding the scope of the work	
	Overall feasible	
Interestingness		
	It interests me	The researcher should be able to find interested collaborators easily in the field; consider using *binary options* instead of a 5-point Likert scale
	I will pursue it if possible.	
	Overall an interesting idea	
Novelty		
	Leads to innovation in medical practice	To test if the hypothesis has the potential to lead to innovations in each of these aspects (i.e., subitems), evaluated on a 5-point Likert scale
	This leads to new methodologies for clinical research	
	It may alter previous findings	
	Leads to novel medical knowledge	
	This leads to new findings, which can be incremental	
	Overall novel	
Potential benefits and risks		
	Significant benefits	To test if the hypothesis has the potential to provide significant benefits over risks to stakeholders; consider using *binary options* instead of a 5-point Likert scale
	No or tolerable risks	
	The overall benefits outweigh the risks	
**Significance**		
	Addressing established medical needs	To test if the hypothesis has the potential to have an impact on each of these aspects (i.e., subitems), evaluated on a 5-point Likert scale
	Impact future direction of the field	
	Impact on the target population	
	Impact the cost and benefit	
	Overall significant	
Testability		
	It can be tested in an ideal setting	The hypothesis can be tested, regardless of feasibility, and evaluated on a 5-point Likert scale
	Adequate number of patients to choose from	
	Overall testable	
**Validity**		
	Scientific validity	The hypothesis is scientifically and clinically valid, evaluated on a 5-point Likert scale
	Clinical validity	
	Overall valid	

Note: Validity, significance, and feasibility, denoted in bold, were used in the brief version of the instrument to conduct gateway evaluations for all the hypotheses generated in the study

In experimental evaluation 1, the experts’ evaluation scores for the 19 hypotheses across the ten dimensions were averaged, and none of the ten dimensions could achieve a moderate ICC coefficient (> 0.50). ICC, intraclass correlation coefficients (two-way mixed effects for absolute agreement), was used to measure inter-rater agreement among the seven experts. According to Koo and Li’s guidelines [37], acceptable inter-rater reliability should have at least 0.5 or higher ICC values. Therefore, experimental evaluation 2 was conducted, validity was set as a screening item, and one highest and one lowest rated examples of hypotheses from experimental evaluation 1 were provided in the instructions of experimental evaluation 2 to help expert panel members calibrate their ratings.

In the experimental evaluation 2 result analysis, the results of the screening item were checked first. The valid sample size included 17 hypotheses (out of 30) in experimental evaluation 2. Then, the inter-rater agreement of the 17 hypotheses was checked using ICC analyses. Half of the ten dimensions achieved a moderate ICC value (0.50–0.75), indicating an acceptable level of absolute agreement on the ratings among the seven experts [38]. Based on the ICC results, qualitative evaluation of the ten dimensions, and our own experience, a decision was made to retain three measures (i.e., validity, significance, and feasibility) for a shortened version of the evaluation instrument.

**Table 2:** Example of hypotheses and evaluation results using the 3-item instrument

Hypothesis	Three evaluation items	R1	R2	R3	R4	R5	R6	R7	Item means	SD	Hypothesis mean ± 95% confidence interval
H1	Validity	5	5	5	3	4	3	4	4.14	0.83	4.0 ± 0.35
	Significance	4	4	4	3	3	2	4	3.43	0.73	
	Feasibility	5	4	4	4	5	5	4	4.43	0.49	
H2	Validity	NA	3	4	3	3	3	1	2.83	0.90	2.64 ± 0.52
	Significance	NA	3	4	4	2	3	2	3	0.96	
	Feasibility	NA	3	2	3	2	1	1	2	0.82	

Note: **Hypothesis 1**: Patients who have hypertension between 2005 and 2015, do hypertension patients have a higher obesity morbidity rate (ICD9 codes: 27801) in 2015 than in 2005? **Hypothesis 2**: Whether the changes in packed food consumption caused an increase in diabetes (ICD9 code: #250) from 2005 (case counts: 774) to 2015 (case counts: 1281) at the zip code level? R1: reviewer 1; NA: not applicable (i.e., an evaluator cannot assess the item, a hypothesis is invalid, and all following items are not evaluated)

We averaged experts’ ratings for each item and compared the hypothesis means from the 3-item instrument (Appendix 4) and those from the 10-item instrument using a paired *t*-test. The results indicated no significant difference between the two sets of ratings (t = 1.74, *p* = .13), which supported the statement that the two instruments performed without significant differences. Figure 3 shows the 10-item evaluation instrument used for experimental evaluation 2, including the highest and the lowest-rated hypotheses as examples.

## Discussion

### Interpretation of the results

Hypothesis generation is a highly sophisticated cognitive process. Not all information used during the processes is a conscious or explicit choice. Our study explored the process of scientific hypothesis generation using the same clinical datasets to determine whether a secondary data analytic tool could facilitate the process. Establishing the evaluation metrics was the first step and was the critical foundation for the overall study and understanding of the entire process. Comprehensive and objective measures were given more weight during the development of the metrics. In our studies, the clinical researchers generated a few to over a dozen hypotheses within two hours [12, 13]. However, not all hypotheses were of high quality. Therefore, it was not conducive to using the experts’ time to comprehensively evaluate each hypothesis generated during the study sessions.

Furthermore, using the entire set of metrics, including all items and subitems, to evaluate each generated hypothesis may be unnecessary. Thus, we used “gateway” evaluations as a filter to identify the higher-quality hypotheses. The experts can determine the higher-quality hypotheses more carefully, thoroughly, and comprehensively during the comprehensive evaluation. Therefore, validity was used as a screening item, and the “not a hypothesis” option was added in the initial assessment, enlightened by the experimental evaluation 1 results.

The results of experimental evaluation 2 aided in determining a brief evaluation instrument with the 3 items used to evaluate the rest of the hypotheses generated by the participants during the gateway evaluation (Figs. 1 and 2). From the ICC analysis in experimental evaluation 2, feasibility, testability, and clarity have the highest ICC values among the ten items, which indicates higher agreement on these dimensions among the expert panel members. However, empirically, we highly prioritize validity, significance, and novelty for clinical research projects. Combining our experience and the statistical testing results, we developed two options: validity, significance, and feasibility; validity, significance, clinical relevance, and feasibility. The testing results indicated that both were valid options. Thus, we determined the 3-item evaluation instrument for easier operational purposes. We used our experience and statistical testing results to guide decision-making.

Meanwhile, we noticed negative ICC values in ethicality, potential benefits and risks, and interestingness. The results indicated that reaching a consensus on these items might be challenging. We recommend that these three items change to a binary (yes/no) category instead of a 5-point Likert scale to simplify the evaluation and improve the agreement among the evaluators.

During the external validation, one major result was to add “not applicable” as an option to the evaluation instrument under each item and subitem. Considering the different backgrounds of expert panel members, this additional option helped them to simplify the evaluation process. Comparing the statistical results, we noticed a significant improvement in experimental evaluation 2, mainly due to the examples of the highest and the lowest-rated hypotheses, which might assist evaluators in calibrating their expectations. Furthermore, we reminded the evaluators that some statements were not hypotheses, i.e., we used validity as a screening item. The experimental evaluation 2 results are based on 17 valid hypotheses. The 13 invalid hypotheses have three or more expert panel members who evaluated them as 1 (the lowest score) in the dimension of validity.

Although the evaluation of a particular hypothesis by an expert can be subjective, we used examples of the highest and the lowest-rated hypotheses to assist experts in calibrating their expectations more accurately. The inclusion of seven expert members balances the subjectivity and provides a more consistent evaluation using the same instrument, which aligns with publications in the field [39]. In addition, we used objective measures, e.g., the number of hypotheses generated and the average time spent on each hypothesis, and randomized the hypotheses during the assessment. These strategies helped the expert panel to provide more consistent evaluations and allowed us to accurately conclude the quality of the hypotheses. The two examples and their rating results in Table 2 elaborate on the process. According to the results, reviewers rated hypothesis 1 (hypertension-obesity connection) higher in validity, significance, and feasibility than hypothesis 2 (diabetes and packed food consumption). The rating differences were larger in ratings of validity (4.14 versus 2.83) and feasibility (4.43 versus 2), although significance ratings were much closer (3.43 versus 3). For hypothesis 2, the lower feasibility rating might be related to difficulties obtaining dietary information over time.

We want to emphasize a critical point for peers who may use our metrics and instruments: we strongly encourage a pilot evaluation to test validity before the metrics are used. We hope our methods in this manuscript will be used as an example of how such calibration can be conducted instead of using our example as a one-test-fits-all scenario to assume our brief version can be applied to *all* potential assessments. We provided a relatively comprehensive evaluation of items and subitems and hoped users could choose needed items from the comprehensive pool for their purposes; however, such selection should be validated via testing with their datasets.

### Strengths and weaknesses of the study

The most obvious strength of our study is we developed and validated the metrics and instruments for evaluating hypotheses in a clinical research context. After several failed literature searches for existing metrics, scales, or instruments, we decided to develop and validate a tool for our study and the broader clinical research community. While preparing this section, we reviewed all 84 records similar to this manuscript (as a preprint) in PubMed, with no publication about similar purposes, which further confirmed no such metrics/instruments exist. Therefore, this paper is the first to present the metrics and instruments to assess scientific hypotheses for clinical research projects. We validated and tested them, although most evaluation items and subitems originated from existing textbooks and papers about clinical research and clinical trials. The metrics and the instruments are systematic and convenient tools for the clinical research community.

We recognize the crucial role of social and ecological accountability in modern practices. It entails taking responsibility for our products’ impact on society, including communities and the environment. We do need to emphasize ethical standards and adhere to certain social and cultural standards. We hope to address these issues in our future research.

### This study within the literature context

Although we had several failed literature searches before we initiated our study, some similar studies are worth mentioning. The first paper is a highly cited methodological instrument development and validation paper that was published in 2003 by Slim et al., a French group [40]. That study comes up with a 12-item list (e.g., stated aim, inclusion criteria, loss to follow-up not exceeding 5%) for methodological quality evaluation for non-randomized studies in surgery [40]. Our study used very similar principles during the instrument development and validation. However, we focused on very different evaluation dimensions (quality of hypotheses in clinical research, e.g., validity and significance) from theirs (a checklist for reporting the quality of nonrandomized studies). A group in the UK developed a checklist for research to describe health service interventions [41]. The checklist includes patient group, organization, location, workforce and staffing, and other context information [41]. A clear difference between their study and ours is that we aim to evaluate the quality of the scientific hypotheses in clinical research projects despite the overlap between our overall goals. There are other efforts to develop and evaluate the patient self-assessment instruments to assess primary care quality [42], instruments for diabetes health literacy scales [43], and instruments to assess the quality of clinical care guidelines [44] and drug studies [45]. An additional example includes reporting on the quality of randomized control trials [46]. Although these studies are remotely relevant to our work, they are different from our study. We compare our study with theirs to put our metrics and instruments into context and emphasize the unique contributions of our study.

## Conclusion

The metrics and instruments developed in this study can benefit clinical researchers in evaluating their hypotheses more comprehensively, consistently, and efficiently before launching a research project, as well as providing valid instruments for the peer review process in clinical research. Our results provide an evidence-based brief version (validity, significance, and feasibility) and a comprehensive version of the evaluation items (validity, significance, feasibility, novelty, clinical relevance, testability, clarity, ethicality, potential benefits and risks, and interesting to others) to assess the quality of clinical research hypotheses. The metrics can be used to standardize the process and provide a consistent tool for this highly sophisticated cognitive process.

## Electronic supplementary material

Below is the link to the electronic supplementary material.

Supplementary Material 1

Supplementary Material 2

Supplementary Material 3

Supplementary Material 4

## Data Availability

All data and instruments were included in the manuscript. Please contact the corresponding author if additional data are needed, and the final decision will be made on a case-by-case basis.
