# Supplementary material for: Development, validation, and usage of metrics to evaluate the quality of clinical research hypotheses
Source: BMC Med Res Methodol. 2025 Jan 16;25:11. doi: 10.1186/s12874-025-02460-1 (PMC11737058; doi:10.1186/s12874-025-02460-1)
Supplement: Supplementary file 1 — Supplementary Material 1 [file 12874_2025_2460_MOESM1_ESM.pdf]

Appendix 4: Evaluation instrument of 3 items without subitems to evaluate the scientific hypotheses in clinical research
3-item clinical hypotheses evaluation

Overall guidance for the survey:

- If you hover the mouse over each dimension, such as Validity, you will see the definition and examples of the dimension.
- Example hypothesis 1 with an overall 4.0 rating: Given the COVID-19 pandemic, more patients use telemedicine for their healthcare services. We hypothesize that after COVID-19, more patients would like to have telehealth visits
- Example hypothesis 2 with a 4.0 rating: Patients who have hypertension between 2005 and 2015, do hypertension patients have a higher rate of morbid obesity (ICD9 codes: 27801) in 2015 than in 2005?
- Example hypothesis 3 with an overall 2.8 rating: Some kind of respiratory disorder may lead to the COPD
- Example hypothesis 4 with 2.47 rating: I hypothesize that the different infant mortality rates among private health insurance beneficiaries and Medicaid beneficiaries will help us infer the social-economic status differences between the two groups to identify the key factors contributing to the higher infant mortality rate.
- **To combine 2005 and 2015 data sets and treat the combined data set as a whole and look at it more closely** is not a hypothesis; if a statement is ***not a hypothesis***, please select the lowest score for validity and ***no other dimensions need to be evaluated for that hypothesis***.

Hypothesis 1: To compare different states in the USA if the incidence of 5859 (ICD9 code: chronic kidney diseases, unspecified) is correlated to kidney transplantation surgeries. If there are differences, what causes these differences? Donors? Surgeons? Or other reasons.

Please provide an overall score from 1 (the lowest) to 5 (the highest) on each dimension for the hypothesis.

	1	2	3	4	5	Not Applicable
Validity						☐
Significance						☐
Feasibility						☐

Powered by Qualtrics
